# Good rate of satisfaction but suboptimal clinical outcome at long‐term follow‐up in a large series of patients who had operative stabilization of the deltoid ligament of the ankle

**DOI:** 10.1002/ksa.12459

**Published:** 2024-09-19

**Authors:** Michael Rindom Krogsgaard, Henrik Palm, Kenneth Obionu, Yvette Astrup, Naja Bjørslev Lange, Tobias Haak, Christian Dippmann

**Affiliations:** ^1^ Department of Orthopedic Surgery Copenhagen University Hospital Bispebjerg Copenhagen Denmark

**Keywords:** ankle instability, ankle pain, deltoid ligament injury, FAAM, medial ankle ligament injury, reconstruction

## Abstract

**Purpose:**

To report results following deltoid ligament reconstruction in a large series of patients.

**Methods:**

For consecutive patients who had a medial ankle ligament stabilization 2010–2018 information from their medical files was registered, and they were invited for follow‐up in 2021–2022. They answered questions about satisfaction with the treatment and current symptoms, and they completed the Foot and Ankle Measurement (FAAM) questionnaire.

**Results:**

Of the 503 patients, 342 (68%) had a history of trauma to the ankle and 114 (23%) had previous ankle surgery. 67% had other procedures (besides synovectomy) simultaneous to medial ligament reconstruction. 269 patients (54%) responded to the invitation for follow‐up. For 182 (71%) of the responders, the operation solved their ankle problems. 163 (63%) were satisfied with the surgery. 192 (71%) would repeat the operation. 173 (67%) had pain in the ankle during the past week, and 86 (50%) of these were not able to run. FAAM ADL‐scores were significantly higher than in a mixed group of ankle/foot patients but not normal.

**Conclusion:**

The relatively high degree of satisfaction despite suboptimal clinical results may reflect the complex nature of the deltoid ligament insufficient ankle. It is concluded that repair or reconstruction of the deltoid ligament is only performed in patients reporting ankle instability and with peroperatively demonstrated medial instability and pathology to the ligament.

**Level of Evidence:**

Level III.

**Abbreviations**
ADLs: activities of daily living
AOFAS: American Orthopaedic Foot and Ankle Society
CARS: Copenhagen Ankle Range of Motion Scale
FAAM: Foot and Ankle Measurement
PROM: patient‐related outcome measure
ROM: range of motion

## INTRODUCTION

The deltoid ligament provides stability of the medial side of the talocrural joint and the foot. It is a complex anatomical structure [20]. Injuries of the medial ankle ligaments are classified into three types according to location [10, 19].

The traditional opinion that lesions to the medial ankle ligament complex are rare [9] has been challenged, as up to 40% of patients with an ankle fracture also have a deltoid ligament injury [18, 25], and more than 70% with lateral ankle instability have pathology of the deltoid ligament on magnetic resonance imaging (MRI) [5]. In patients with chronic ankle instability following lateral ankle ligament injury, 40% also had arthroscopically verified lesions of the deltoid ligament [11]. However, there is no consensus algorithm for when surgical intervention might be indicated for this pathology.

Patients with deltoid ligament instability report of medial ‘giving‐way’, pain, and swelling [10, 18]. For the diagnostic process, a history of a relevant trauma, clinical examination, radiographs, MRI and direct arthroscopic visualization of the injury are all important elements [12, 15].

Medial ankle instability can lead to chronic instability, progressive deformity, and ankle joint osteoarthritis. Reconstruction of the medial ligaments can be indicated, but there is limited evidence in the literature for the treatment strategy and results [20].

Reconstruction with Fiberwire® was standard at the Department of Orthopaedic Surgery, Copenhagen University Hospital Bispebjerg, and Frederiksberg for a 12‐year period. The hypothesis of the current study was that the results of this treatment strategy would be comparable to what had been reported in few, small series on deltoid ligament reconstruction. The results of follow‐up on this treatment cohort are presented in the current manuscript.

## MATERIALS AND METHODS

Permission for this study was obtained from the registered study authorities of The Capital Region in Denmark (Journal number R‐21022743), from the management of Copenhagen University Hospital Bispebjerg (WZ 21033122), and from the data protection authorities of the Capital Region in Denmark (P‐2021‐425).

Consecutive patients who had a medial ankle ligament reconstruction within the period 2007–2018 were retrospectively identified in the operation databases for Orthopaedic Department at Copenhagen University Hospital Bispebjerg and Frederiksberg. Due to implementation of a new digital healthcare system in 2016, medical files earlier than 2010 were not digitally accessible, and therefore this study included patients operated after 1 January 2010. The data listed in Supporting Information S1: Table 1 were extracted from the medical files.

The operative strategy was based on the á la carte principle, meaning that all relevant pathologies were treated. As the indication for deltoid ligament reconstruction seemed more liberal than in other studies in literature—based on the large cohort—the clinical usefulness of this follow‐up study was to determine whether this strategy is advantageous with good clinical outcomes or whether the indication should be narrowed. Three foot/ankle subspecialist surgeons performed all operations, principally starting by testing for ankle stability while patient was under anaesthesia. The deltoid ligament and any additional pathology of the ankle joint were visualized by arthroscopy. An arthroscopically verified lesion of the deltoid ligament was described in all cases where reconstruction was performed. A curved incision was made over the medial malleolus, and dissection was performed on the bony surface. Onto two sets of drill holes in the medial malleolus, the torn‐off or degenerated ligament component was reattached with a non‐absorbable suture (Fiberwire®). The subcutaneous tissue was closed with Vicryl® and the skin with nylon. In case of concomitant lateral instability, the lateral side was stabilized, in most cases by a Broström procedure [2]. The patients wore a walker boot for 6 weeks with or without weight‐bearing (depending on pathology). Physiotherapy was not offered routinely.

All patients were invited in September–November 2021 to report how their ankle was doing via the digital platform (www.e-Boks.dk) that is used by health authorities, government, and many public authorities and private companies in the communication with persons in e‐Boks.dk. After accepting the invitation, they could log‐in via a digital secure link, using Redcap®, to answer specific questions related to the period after the operation, a PROM (patient‐related outcome measure), an ankle ROM (range of motion) scale, and a number of anchor questions related to the degree of satisfaction with the treatment (Supporting Information S1: Table 1).

Based on an analysis of 17 foot‐ and ankle‐PROMs [6], the most adequate was identified as the Foot and Ankle Measurement score (FAAM) which assesses the physical function of individuals with lower leg, ankle, and foot musculoskeletal disorders [6]. The PROM was translated by a bilingual panel, and wording was secured by a lay panel. It was culturally adapted and construct validated in Denmark [21]. Based on confirmatory factor analysis and Rasch analysis of data, the original 29‐item version of the PROM was reduced to 22 items in two domains (activities of daily living [ADLs] and Sports) plus two visual analogue scales to describe activity in general and during sports [21]. In connection with this construct validation of the questionnaire patients with various conditions in the ankle and foot completed FAAM‐Denmark, and these data were used for comparison with the current cohort.

ROM in the operated and the unoperated ankle was reported through the Copenhagen Ankle Range of Motion (ROM) Scale (CARS) (Personal communication).

Patients who did not react to the invitation were not reminded about the survey, and 8 weeks after the last patient had been contacted, the Redcap® link was inactivated. The three surgeons who had performed the operations were working at other hospitals and were not involved in the follow‐up.

To establish a normal material of FAAM responses, 50 employees at the Department of Orthopaedics with no injury or symptoms in the ankles or feet completed the FAAM‐Denmark (Control).

### Statistical analysis

The descriptive presentation was based on data registered in Excel and calculated in ‘R’ version 2023.06.2+561: mean, standard deviation, and confidence intervals. The primary outcome for statistical evaluation was the FAAM score in each of the two domains ADL and Sports. The minimal clinically important difference (MCID) for FAAM for patients with deltoid ligament instability is unknown, but for the current cohort, it can be estimated mathematically as ½ SD [4], meaning that MCID for FAAM‐ADL and FAAM‐Sports is 6.9 and 4.3 points, respectively. The differences between groups were tested by *t* test, and the significance level was set at 0.05. To be able to demonstrate a difference exceeding the MCID for FAAM‐ADL with alpha 0.05 and beta 80%, the necessary number of patients is 32 and for FAAM‐Sports 31 patients.

Data based on the medical files and MRI scans were available for all patients.

## RESULTS

After excluding dead and emigrated patients, the cohort included 503 patients (524 ankles: 234 left, 248 right and 21 bilateral). There were 309 females and 194 males. The median age was 45 years (range: 20–80).

All operations had been performed by three foot/ankle surgeons: one operated on 364 patients, one on 119, and one on 20. The time interval between the operation and the current follow‐up was median of 6.8 years (range: 3.1–11.9 years)

There were 342 patients with a history of ankle trauma on the affected side (fracture or sprain), 68 patients had no history of trauma, and for 93 patients no information regarding trauma was available. Previous ankle surgery had been performed in 114 patients (Supporting Information S1: Table 2), and 79 patients had previously participated in a physiotherapy programme without satisfactory effect. The presence of subjective ankle instability and medial ankle pain as indicated by the patients, and clinical, objective instability/tenderness is shown in Table 1.

**Table 1:** Symptoms and clinical findings according to the medical files for 503 patients operated with medial stabilization of the ankle.

	Yes	No	No information
Subjective ankle instability	204 (50.5%)	200	99
Objective ankle instability	139 (36.3%)	243	121
Subjective medial ankle pain	130 (38.1%)	211	162
Objective medial ankle tenderness	190 (47.9%)	175	138

*Note*: Percentages relate to patients with available information.

The surgeon evaluated that the MRI for 447 patients was showing pathology to the deltoid ligament. In contrast, according to the radiological description of the MRI, there was only pathology of the deltoid ligament in 154 cases. For the 388 MRI scans that had been evaluated by a surgeon as well as a radiologist, the surgeon and radiologist had identical evaluations in relation to the deltoid ligament in 153 cases (39.4%) and in 142 cases (36.6%) they both described pathology of the ligament.

Leaving arthroscopic synovectomy out, 165 ankles (32.9%) had reconstruction of the medial ligament as the only procedure. Additional surgical procedures were performed in 337 patients (67.1%) during the index operation (Supporting Information S1: Table 3).

A total of 120 ft. (22.9%) were subsequently re‐operated at the index hospital. Superficial infection treated with antibiotics was reported in 25 ft. (4.8%), and deep infection was treated surgically in four feet (0.8%). The Fiber‐wire® was removed in 65 cases (12.4%), and arthroscopic debridement, removal of osteophytes, osteotomies, and other operations not related to the index operation were performed in 55 cases (10.5%). There was no case of Chronic Regional Pain Syndrome. Some patients were subsequently operated at other hospitals, but information on this surgery was not available.

When the Red Redcap® Cap link was closed, a total of 269 (53.8%) patients had responded to the invitation. Of these, 258 completed the questionnaire while 11 indicated that they did not wish to participate in the follow‐up study. The following results relate to the 258 respondents, who had a mean age of 45 years (range: 20–79). Answers to questions on satisfaction and pain are reported in Table 2.

**Table 2:** Post‐operative responses regarding satisfaction and pain in the operated ankle from 258 patients who had a deltoid ligament reconstruction.

Did the surgery solve your ankle problems?	
Yes, completely	55 (21.4%)
Yes, to some extent	127 (49.2%)
No change	28 (10.9%)
No, symptoms became worse	48 (18.5%)
Are you satisfied with the results of the operation?	
Yes, very satisfied	74 (28.7%)
Yes, partially satisfied	89 (34.6%)
No, partially dissatisfied	45 (17.3%)
No, very dissatisfied	42 (16.5%)
Cannot be determined	8 (3%)
Prior to your operation, if you could know the current results of your operation on your symptoms, would you still choose to be operated?	
Yes	163 (63%)
Maybe	29 (11.3%)
No	53 (20.6%)
Cannot be determined	13 (5%)
Have you experienced any form of pain in your ankle during the last week?	
Yes	173 (67.2%)
No	85 (32.8%)
If yes to the pain question: Which activities are associated with pain in your ankle?*	
Walking	145 (83.8%)
Ascending/descending stairs	95 (54.9%)
Running	55 (31.8%)
I cannot run at all	86 (49.7%)

*Note*: Percentages refer to the 258 patients, except * which refers to patients with pain.

FAAM‐Denmark scores at follow‐up and from a mixed group of ankle/foot patients and 50 healthy persons are reported in Table 3. For FAAM‐ADL and FAAM‐Sports, the scores for the current cohort were significantly higher than scores from the mixed group of patients (*p* < 0.05) but significantly lower than scores from a normal population (*p* < 0.05).

**Table 3:** The subjective outcome as measured with the FAAM score in the group of 258 responders who had a deltoid repair, a group of 206 patients with various foot‐ and ankle conditions who responded to the FAAM questionnaire in relation to construct validation of the score [18], and 50 normal persons.

	Deltoid operated (*n* = 258)	Mixed foot/ankle patients (*n* = 206)	Normal cohort (*n* = 50)
FAAM‐ADL	43.6 (13.8; 37.2–50.0)	19.5 (9.2; 15.2–23.7)	58.1 (3.7; 56.4–59.8)
FAAM‐Sport	12.0 (8.5; 8.1–15.9)	27.8 (19.2; 19.0–36.7)	23.8 (5.7; 21.2–26.5)
FAAM ADL %	72.1 (25.9; 60.1–84.0)	‐	92.9 (16.8; 85.2–100.7)
FAAM Sport %	53.1 (32.3; 38.1–68.0)	‐	90.7 (15.9; 83.4–98.1)

*Note*: Values are means with standard deviation and confidence intervals in parentheses. For FAAM‐ADL and FAAM‐Sports, the scores for Deltoid‐operated patients were significantly (*p* < 0.05) higher than scores from the group with mixed conditions and significantly lower (*p* < 0.05) than scores from normal persons.

Abbreviations: ADLs, activities of daily living; FAAM, Foot and Ankle Measurement.

The subjective outcomes were independent of whether the radiologist had evaluated that there was pathology of the deltoid ligament on MRI or not (*p* > 0.05).

The 238 patients who had a unilateral operation indicated whether ROM in the operated ankle compared to the unoperated side was identical (51 cases), reduced (175 cases = 74%) or larger (10 cases). ROM reported by the CARS confirmed this distribution (Table 4).

**Table 4:** Self‐reported ROM, evaluated with the Copenhagen ankle ROM scale by 239 patients with unilateral deltoid ligament surgery.

	Extension (down)	Flexion unloaded (up)	Flexion loaded (up, standing)
Operated side	4.9 (1.2; 4.3–5.4)	2.5 (0.6; 2.2–2.7)	3.1 (0.7; 2.8–3.4)
Unoperated side	5.5 (0.9; 5.1–6.0)	2.8 (0.5; 2.6–3.0)	3.6 (0.6; 3.3–3.9)
Better ROM (%)/a	9 (4%)/	8 (3%)/	2 (1%)/
Same ROM (%)/	130 (54%)/	150 (63%)/	115 (48%)/
Reduced ROM (%)	100 (42%)	81 (34%)	122 (51%)

*Note*: Values are means with standard deviation and confidence intervals in parentheses. Extension was described by six pictures, unloaded flexion by three pictures, and loaded flexion by four pictures, and the number relates to the picture (personal communication).

Abbreviation: ROM, range of motion.

a Number of patients who had indicated on the pictorial assessment a better, identical, or reduced ROM in the operated ankle compared to the normal ankle. Percentages in parentheses.

## DISCUSSION

The main result of this long‐term follow‐up after deltoid ligament reconstruction was that despite 67% having pain in the ankle (and only 50% of these patients being able to run), as well as 74% reporting reduced ROM, 63% were satisfied with the operation, 70% felt that the operation had solved the problem they had been admitted for (fully or in part), and 74% would still choose the operation given the knowledge they have today. It can be debated whether this is a good result.

In a series of 52 patients with a superficial deltoid ligament insufficiency, Hintermann et al. [12] performed repair of the deltoid ligament in all cases, while it was also necessary to repair the spring ligament in 24% and the lateral ligaments in 77%. The clinical results in this series were considered as ‘good to excellent’ in 90%, and it was concluded that the strategy to manage deltoid ligament injuries and at the same time treat additional pathology of the ankle leads to favourable results.

In a series of 17 patients with chronic deltoid ligament instability and repair of the deep as well as the superficial parts of the ligament and synovectomy and cartilage debris removal, when necessary, malalignment of the hindfoot was reduced, and the mean American Orthopaedic Foot and Ankle Society (AOFAS) ankle‐hindfoot score increased, and based on this, 10 patients were rated as excellent, six as good, and one as fair. Pain was reduced in all patients [24]. However, interpretation of the subjective results of the study can be difficult, as the AOFAS ankle‐hindfoot score has no proven content validity, because no patients had been involved in its development [7].

In 13 patients with rotational ankle instability, an ‘open book’ tear of the deltoid ligament was demonstrated and repaired during arthroscopy simultaneously with lateral ankle ligament repair. The AOFAS score increased [22]. This was also the case for 29 patients, who had lateral and medial ligament repair for rotational ankle instability [16], but more than half of the patients also had other procedures. In a comparative study between repair and non‐repair of the deltoid ligament in 50 patients treated for rotational ankle stability with lateral reconstruction, there was no difference between the groups in patient‐reported outcome scores [14].

After Broström's operation for lateral ankle instability [2], at least 88% are reported to be satisfied with surgery [17], even though there is a 13%–35% prevalence of persistent pain [1]. Up to 92% of patients report good or excellent outcomes with no recurrent sprains or difficulty walking up or down hills or on uneven ground [3].

Therefore, the results in the current cohort are inferior to outcomes reported in literature after lateral and medial ligament reconstruction.

Based on the preoperative medical information, less than 40% of the patients complained of medial ankle pain and only about half had tenderness in this area. Only half of the patients complained of ankle instability preoperatively and less than 40% were clinically unstable medially, so post‐operative pain may be caused by overtightening of the superficial deltoid ligament in patients who were not unstable. That reconstruction of the deltoid ligament can result in over constraint has recently been demonstrated in a cadaver study [23].

Lesions to the deltoid ligament were confirmed by direct visualization in all cases as recommended [10, 12], but it is suggested that decisions on repair of the deltoid ligament must be based on the combined presence of confirmed rupture of the ligament, patient's subjective feeling of instability, and clinical instability documented peroperatively as a medial clear space in the ankle joint, showing as a triangle between tibia, the medial malleolus and talus) during fluoroscopy while applying valgus to the hindfoot (the talar tilt test).

The outcome may also reflect that two thirds of the patients in the cohort had other concomitant pathologies that were addressed surgically. This is also reported to be the case in the other existing series after deltoid ligament reconstruction [12, 13, 14, 16, 22, 24], and this limits the ability of the studies to evaluate the effect of deltoid ligament surgery, as the contribution of each surgical procedure cannot be separated in the final outcome. Therefore, the relevance of reconstruction of the deltoid ligament can only be evaluated in a randomized, controlled study.

While the FAAM ADL domain scores were substantially higher than in a group of patients with various ankle‐ and foot conditions, although not normal, the FAAM sports domain scores were remarkably low in the current group. However, this is not surprising, as at least 33% were not able to run at follow‐up, and many of the activities in the sports domain are based on running.

There is a normal variant of the deltoid ligament showing congenital absence of the anterior tibiotalar ligament on MRI images in half of asymptomatic subjects [19], and this could be falsely interpreted as injury to the ligament. Combined with the finding that the subjective post‐operative outcome was independent of the MRI evaluation of pathology to the deltoid ligament, this supports that the basis for final diagnosis of medial ankle‐instability should also include a diagnostic arthroscopy and perioperative documentation of instability by x‐ray [8].

A reoperation rate of at least 23% is very high, and half of the cases were removal of the Fiberwire®. The use of absorbable suture material might eliminate a significant proportion of the reoperations.

This is a follow‐up based on a retrospective cohort, meaning that information about the preoperative condition is of varying quality. The operation, however, was standardized, and patients were consecutively identified. The cohort is almost 10 times larger than the series reported in the literature to date. Unfortunately, the follow‐up rate was only 54%, even though it is not obvious how it might potentially have affected the results.

## CONCLUSION

The relatively high degree of satisfaction in this cohort despite suboptimal clinical results and a high reoperation rate probably reflects the complex nature of pathology in the deltoid ligament insufficient ankle.

## AUTHOR CONTRIBUTIONS

Michael Rindom Krogsgaard, Henrik Palm and Kenneth Obionu produced the idea and protocol for the study and obtained all necessary permissions. All authors participated in the development of the questionnaire. Christian Dippmann produced the Redcap® database and retrieved data from it. Kenneth Obionu, Naja Bjørslev Lange, Tobias Haak and Yvette Astrup retrieved information from the medical files and the MRI scans. Kenneth Obionu did the statistical analyses, and all authors participated in the interpretation of the data. Michael Rindom Krogsgaard and Kenneth Obionu drafted the manuscript, which was commented on and finally accepted by all authors.

## CONFLICT OF INTEREST STATEMENT

The authors declare no conflicts of interest.

## ETHICS STATEMENT

An application for ethical approval for this study was waived by the ethical committee of the Capital Region in Denmark as there was no intervention involved. Permission to obtain information from the medical files and to register patient data in a database for the study was obtained from the regional health authorities. Permission to retrieve information from the medical files and to register patient data in a database for the study was obtained from the regional health authorities.

## Supporting information

Supporting information.

## Data Availability

The anonymized data set is available at reasonable request, provided that a data‐sharing contract is established with relevant authorities.
