# Supplementary material for: Good rate of satisfaction but suboptimal clinical outcome at long‐term follow‐up in a large series of patients who had operative stabilization of the deltoid ligament of the ankle
Source: Knee Surg Sports Traumatol Arthrosc. 2024 Sep 19;33(4):1524–30. doi: 10.1002/ksa.12459 (PMC11948160; doi:10.1002/ksa.12459)
Supplement: Supplementary file 1 — Supporting information. [file KSA-33-1524-s001.docx]

Supplementary files in relation to the manuscript “Good rate of satisfaction but suboptimal clinical outcome at long-term follow-up in a large series of patients who had operative stabilization of the deltoid ligament of the ankle.”

Supplementary table 1:

Side (right/left/both) – in case of both ankles, two cases under the same study number were opened

Age at the time of surgery

Gender

Trauma (Yes/No)

Previous surgery in the ankle (Yes/No)

Unsuccessful training attempt (Yes/No)

Subjective instability documented (Yes/No)

Subjective medial pain documented (Yes/No)

Objective instability (1 = Yes, medial, 2 = Yes, lateral, 3 = Yes, drawer)

Objective medial tenderness (Yes/No)

Preoperative MRI-scanning (Yes/No)

Medial ankle ligament injury described by MRI radiologist (Yes/No/Not described/No MRI)

Medial ankle instability described by surgeon (Yes/No/Not described/No MRI)

Type of operation on medial side of the ankle (arthroscopy, superficial deltoid ligament repair, deep deltoid ligament repair, spring ligament repair. The ICD procedure code was registered).

Other procedures at the index operation (e.g., surgery for osteochondral lesions, lateral ligament repair, other extra articular tendon repairs/augmentation).

Reoperation (No, Yes (removal of fiberwire), Yes (other operation))

Documented follow-up:

Time from index operation until last primary follow-up (meaning, not as part of the current study)

Subjective instability (Yes/No)

Subjective medial pain (Yes/No)

Objective stability at last follow-up (1 = Stable, 2 = Medial instability, 3 = Lateral instability, 4 = Drawer instability)

Objective tenderness at last follow-up (1 = No, 2= Medial pain, 3= Medial pain + other pain, 4 = Other pain)

Deep infection (No/Yes, treated non-surgically/Yes, treated with surgery and antibiotics)

Nerve injury (No/Yes)

Comorbidities (No/Yes, if yes a description of which)

Supplementary table 1: Information obtained for each patient from the medical files.

Supplementary table 2:

Open procedures:

Tibialis posterior cleaning and reconstruction of spring ligament: 1

Resection of heel spur: 1

Three operations for lateral ankle ligament reconstruction: 3

Peroneal split repair: 2

Open operation for osteochondral lesion on talus: 2

Osteosynthesis of malleolar fracture: 14

Resection of tarsal exostoses: 3

Three operations for talar osteochondral lesion + suralis nerve release: 1

Three release operations because of Achilles tendinitis: 1

Lateral ligament reconstruction (Broström, Evans): 12

Subtalar arthrodesis: 2

Topas burning for heel spur: 1

Operation for mallet toes or hallux valgus: 2

Wedge osteotomy: 1

Removal of Schwannoma: 1

Osteosynthesis tibia with drop foot: 1

Resection of subcutaneous tumor: 1

Achilles tendon elongation: 1

Scopic surgery:

Ankle arthroscopy, unspecified: 8

Arthroscopic ankle synovectomy: 8

Arthroscopic removal of free bodies in the ankle joint: 5

Arthroscopic surgery for osteochondral lesion on talus: 10

Arthroscopic removal of exostosis: 1

Subtalar arthroscopy: 2

Resection of heel spur: 1

Extirpation of os tibiale: 1

Extirpation of a ganglion in sinus tarsi: 1

Unspecified ankle surgery: 27

Supplementary table 2: Previous surgery to the index ankle in 114 patients.

Supplementary table 3:

Removal of ankle osteophytes 68 (13.0 %)

Tendon surgery 62 (11.8 %)

Lateral ligament stabilization 77 (14.7 %)

Debridement of talar cartilage 104 (19.9 %)

Other procedures* 49 (9.4 %)

Supplementary table 3: Procedures performed simultaneous with medial ligament reconstruction in the 524 feet (503 patients) at the index operation, except synovectomy. 165 patients (32.9 %) had no simultaneous procedures.

* For instance, removal of extraarticular osteophytes.
